# Cardiovascular magnetic resonance in the diagnosis of acute heart transplant rejection: a review

**DOI:** 10.1186/1532-429X-11-7

**Published:** 2009-03-12

**Authors:** Craig R Butler, Richard Thompson, Mark Haykowsky, Mustafa Toma, Ian Paterson

**Affiliations:** 1 Division of Cardiology, University of Alberta, Edmonton, AB, Canada; 2 Department of Biomedical Engineering, University of Alberta, Edmonton, AB, Canada; 3 Faculty of Rehabilitation Medicine, University of Alberta, Edmonton, AB, Canada

## Abstract

**Background:**

Screening for organ rejection is a critical component of care for patients who have undergone heart transplantation. Endomyocardial biopsy is the gold standard screening tool, but non-invasive alternatives are needed. Cardiovascular magnetic resonance (CMR) is well suited to provide an alternative to biopsy because of its ability to quantify ventricular function, morphology, and characterize myocardial tissue. CMR is not widely used to screen for heart transplant rejection, despite many trials supporting its use for this indication. This review summarizes the different CMR sequences that can detect heart transplant rejection as well as the strengths and weaknesses of their application.

**Results:**

T2 quantification by spin echo techniques has been criticized for poor reproducibility, but multiple studies show its utility in screening for rejection. Human and animal data estimate that T2 quantification can diagnose rejection with sensitivities and specificities near 90%. There is also a suggestion that T2 quantification can predict rejection episodes in patients with normal endomyocardial biopsies.

T1 quantification has also shown association with biopsy proven rejection in a small number of trials. T1 weighted gadolinium early enhancement appeared promising in animal data, but has had conflicting results in human trials. Late gadolinium enhancement in the diagnosis of rejection has not been evaluated.

CMR derived measures of ventricular morphology and systolic function have insufficient sensitivity to diagnose mild to moderate rejection. CMR derived diastolic function can demonstrate abnormalities in allografts compared to native human hearts, but its ability to diagnose rejection has not yet been tested.

There is promising animal data on the ability of iron oxide contrast agents to illustrate the changes in vascular permeability and macrophage accumulation seen in rejection. Despite good safety data, these contrast agents have not been tested in the human heart transplant population.

**Conclusion:**

T2 quantification has demonstrated the best correlation to biopsy proven heart transplant rejection. Further studies evaluating diastolic function, late gadolinium enhancement, and iron oxide contrast agents to diagnose rejection are needed. Future studies should focus on combining multiple CMR measures into a transplant rejection scoring system which would improve sensitivity and possibly reduce, if not eliminate, the need for endomyocardial biopsy.

## Background

Heart transplantation is a life saving therapy for select individuals with end-stage heart failure[1]. Despite significant advances in anti-rejection therapy, allograft rejection remains a leading cause of mortality with one in four transplant patients dying within five years after surgery [2,3].

### Diagnosis and Screening for Acute Cellular Rejection

Acute cellular rejection is the most common form of heart transplant rejection. Cardiac transplant recipients have between one and three episodes of acute cellular rejection within the first year after transplantation [3]. Moreover, the incidence of cellular rejection requiring treatment is estimated to be 8% and 4% in the first and fifth year post surgery, respectively [3]. Cellular rejection is a host T-cell-mediated reaction to donor antigens resulting in myocardial infiltration with lymphocytes and macrophages, myocardial edema, and myocyte necrosis [4].

Since the early 1970s the gold standard for detection of cellular cardiac rejection has been regular transjugular endomyocardial biopsy [5]. Myocardial tissue obtained from biopsy undergoes histologic grading for severity of cellular rejection as well as immunologic staining to assess for the presence of humoral rejection [6]. Endomyocardial biopsy is uncomfortable for patients and has the potential for rare, but life threatening complications [7,8]. In addition, random biopsy sampling often misses the patchy foci of rejection[9] and there is significant variability in the reporting of histologic specimens [10,11]. Despite these limitations endomyocardial biopsy remains the principle method for rejection screening. It is an important clinical undertaking to find an accurate and less invasive alternative to endomyocardial biopsy for the diagnosis of cardiac transplant rejection.

In the early stages of acute heart transplant rejection the myocardium is inflamed, however, there are often little or no symptoms, nor gross evidence of cardiac dysfunction [4,12,13]. Serial echocardiographic measurements of left ventricular volumes, ejection fraction, wall thickness and mass are too insensitive to screen for transplant rejection in the era of contemporary antirejection therapy [14]. During acute rejection diastolic dysfunction precedes systolic dysfunction [15]. Doppler measures of myocardial diastolic properties such as isovolumic relaxation time (IVRT) [13,16,17], Index of Myocardial performance [18], and Peak Filling Rate [19] have shown correlation to acute rejection though not with uniform consistency [20].

Cardiovascular magnetic resonance (CMR) is the gold standard imaging modality for evaluation of ventricular volumes, morphology, and mass due to superior image quality as compared to echocardiography and nuclear modalities [21-23]. CMR can also measure ventricular diastolic properties such as regional myocardial tissue velocity, strain and rotation [24]. CMR also has proven utility in detecting myocardial inflammation in disease states such as myocardial infarction [25], viral myocarditis [26], Tako-Tsubo cardiomyopathy [27], dilated cardiomyopathy [28], as well as heart transplant rejection in both animal [29-31] and human [16,20,32,33] models. The ability of CMR to characterize ventricular morphology, systolic function, diastolic function, and myocardial inflammation makes it an excellent candidate to non-invasively diagnose and screen for acute heart transplant rejection.

Since the late 1980s there have been many small trials (Table 1) comparing CMR with endomyocardial biopsy in the diagnosis of heart transplant rejection with predominantly positive results [16,20,32-38]. Despite these findings, CMR has not gained widespread use in the surveillance and diagnosis of acute heart transplant rejection. This paper will review the animal and human data supporting the use of CMR for the diagnosis of heart transplant rejection and highlight potential CMR targets for future study.

**Table 1:** T2 values in rejecting and non-rejecting animal models of heart transplantation.

**Trial**	**Animal Model**	**T2 (ms)** **Non-rejector vs. Rejector**	**P value**	**Field Strength**
Walpoth et al 1998	Pig	47 +/- 6 vs. 50 +/- 6	ns	1.5 T

Kurland et al 1989	Rat	35 +/2* vs. 46 +/- 6	< 0.001	1.5 T

Sasaki et al 1987	Dog	36 vs 49	< 0.01	1.5 T

Aherne et al 1986	Dog	42 +/- 5^† ^vs. 66 +/- 8	< 0.01	0.35 T

Tscholakoff et al 1985	Dog	36 +/- 5^† ^vs. 58 +/- 10	< 0.005	0.35 T

Sasaguri 1985	Rat	39 vs. 53	< 0.01	Pulse spectrometer 0.5 T

Huber 1985	Rat	49 +/- 1 vs. 68 +/- 10	< 0.005	Pulse Spectrometer 0.25 T

ns = not significant

* isografts

^†^Non transplant control

### Best Studied CMR Correlates of Heart Transplant Rejection

#### T2 weighted CMR

##### Myocardial T2 signal intensity

Myocardial T2 signal intensity (SI) is influenced by myocardial water content and can clinically detect myocardial inflammation associated with myocarditis [26], Tako-Tsubo cardiomyopathy [27], and acute myocardial infarction[39]. The ability of T2 SI to detect heart transplant rejection has been inconsistent in the literature[29,35]. Aherne et al. showed in a dog model that T2 SI was initially similar between untreated allografts and non-transplant controls, but by day seven, T2 SI was 66% higher in the untreated allograft group compared to controls[29]. Smart et al., and Revel et al. found no difference in T2 weighted SI in patients with biopsy proven rejection compared to those without rejection[34,38]. Notably, Smart et al. did show that serial signal intensities for a given patient increased with biopsy proven rejection and decreased with anti-rejection therapy, however the specificity was only 33%[34]. Alemnar et al. found no association between T2 STIR values and transplant rejection in a group of 40 transplant patients [35]. Despite its utility in other myocardial disease states, T2 signal intensity has shown mixed results in diagnosing heart transplant rejection.

##### Myocardial T2 Quantification

**Figure 1:**
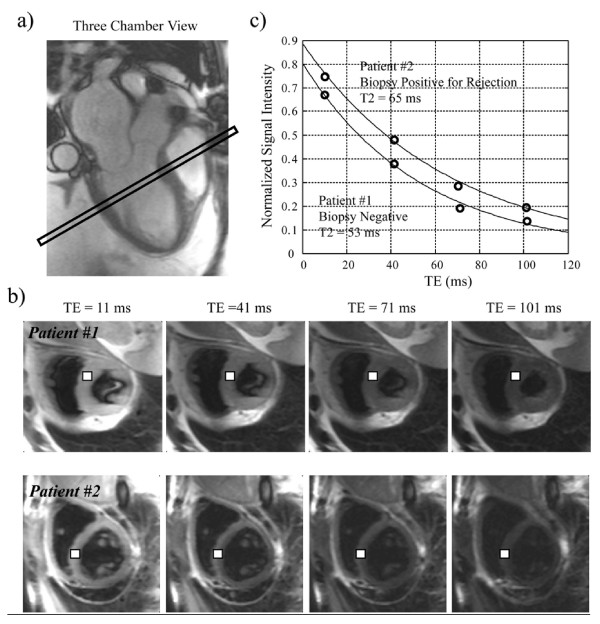
**Comparison of T2 in 2 cardiac transplant patients**. a) Localizing 3 chamber FISP image. b) Axial HASTE images with varying echo times (TE). White square represents septal ROI used to measure signal intensity (SI). c) Plot of SI vs. TE. T2 derived from fitting to curve to an exponential. Patient 1: CMR T2 = 53 ms (normal). Biopsy = no rejection. Patient 2: CMR T2 = 65 ms (elevated). Biopsy = ISHLT grade 2R rejection.

T2 relaxation time is the decay time constant of magnetic signal after an excitatory pulse. T2 relaxation time is calculated by plotting the spin echo signal intensity against varying echo times and is believed to lengthen in proportion to the degree of myocardial edema (Figure 1). Long T2 relaxation times are associated with high tissue water content in models of myocardial infarction[25], myocarditis[26], and animal models of acute rejection [29-31,40] and is therefore a biologically plausible variable to detect human heart transplant rejection.

Normal myocardial T2 relaxation times vary as a function of magnetic field strength and measured values will depend on whether or not an appropriate pulse sequence for quantifying T2 has been used. The last point cannot be stressed enough given that not all T2-weighted pulse sequences are appropriate for obtaining accurate T2 measurements. For this reason, CMR studies generally define their own normal T2 relaxation times from a group of controls and describe abnormal T2 relaxation as more than two standard deviations above the mean [16,20,33].

Since 1985, there have been eleven animal trials evaluating T2 relaxation time and transplant rejection. These trials have used predominantly rat and dog models and applied a variety of imaging platforms, transplant techniques and anti-rejection regimens. Nevertheless, they demonstrated that T2 relaxation times increased with histologic rejection [29,30,40-45] and ex-vivo myocardial water content [29,30,40,41] (Table 1). Furthermore, they also demonstrated that the prolongation of T2 relaxation times observed in transplant rejection could be prevented by the addition of immunosuppressive agents such as cyclosporine [29,42].

There have been eight human trials totaling 302 patients (521 CMR scans) comparing T2 relaxation times to transplant rejection as determined by endomyocardial biopsy (Table 2) [46-48]. Four trials showed significant correlation between T2 relaxation times and transplant rejection (Table 3) [16,20,32,33]. The two trials that did not find an association between T2 and rejection both gated their image acquisition to ventricular systole [36,37] which often leads to signal loss and poor image quality [49,50].

**Table 2:** Human Trials comparing endomyocardial biopsy and CMR in the diagnosis of acute heart transplant rejection

**Author**	**Year**	**n**	**Scans**	**Positive biopsies**	**Age**	**Time from Txplant**	**Delay to CMR**	**Histologic Grading**
Almenar L	2003	40	64	-	51 +/- 13 yrs	13–3725 days	-	Defined*

Marie P.Y	2001	68	123	19	51 +/- 13 yrs	8 +/- 11 mth	<4 days	ISHLT[47]

Marie P.Y	1998	52	52	9	51 +/- 14 yrs	32 +/- 42 mth	< 1 wk	ISHLT[47]

Smart F.W.	1993	8	33	3	-	1–7 mth	<24 hrs	McAlister[48]

Mousseaux E	1993	39	39	7	52 (18–69) yrs	7 d–5 yrs	<48 hrs	ISHLT[47]

Revel	1989	29	33	8^†^	45 (11–60) yrs	3 wk-5 yrs	<3 days	Billingham[46]

Lund G	1988	9	35	2	5 mos – 59 yrs	1–13 wks	<24 hrs	Not stated

Wisenberg G	1987	25	62	16	14–57 yrs	9–107 days	<24 hrs	Defined^‡^

Totals		302	521	75				

- Not available

* ≥1 focus of myocyte necrosis

† rejection defined as Billingham grade 1 or greater

‡ lymphocytic infiltrate with myocytolysis

**Table 3:** T2 values by ISHLT(1990) grade of rejection

**Trial**	**Mean T2 Relaxation Times**				
	**Control**	**Grade 0**	**Grade 1**	**Grade 2**	**Grade 3**

Wisenberg et al (1987)	35 +/- 6	61 +/- 6 (<25 days) 36 +/- 5 (>25 days)	-	62 +/- 6*	-

Lund et al (1988)	47 +/- 8	45 +/- 8	-	69 +/- 8*	-

Marie et al 1998	-	50 +/- 5	-	62 +/- 5*	-

Marie et al 2001	-	50 +/- 5	51 +/- 8	57 +/- 5	65 +/- 8

Mean	41	45		63	-

*≥grade 2 rejection

In an early investigation, Wisenberg et al performed CMR (0.15 T) on ten healthy volunteers to establish normal values for T2 relaxation times and compared them with those obtained from 25 transplant patients scanned within 24 hours of endomyocardial biopsy [33]. Transplant patients were scanned immediately following heart transplantation out to a maximum of 107 days. Patients who were scanned more than 24 days after heart transplantation showed significant correlation between T2 relaxation times and endomyocardial biopsy. All patients who were scanned in the first 24 days post transplantation had elevated T2 relaxation times irrespective of their biopsy results. After 24 days, a T2 relaxation time of > 46 ms (i.e., 2 standard deviations above control T2 relaxation times) achieved a sensitivity of 93% and specificity of 96% for detecting rejection. A second control group of patients undergoing non-transplant thoracotomy displayed T2 relaxation times that were not different from healthy volunteers. This suggests that T2 relaxation times are not able to discriminate rejecting and non-rejecting allografts in the peri-operative period. It also suggests that there are early causes of inflammation in heart transplantation not related to rejection. Pereira et al. have shown that in the first week post heart transplantation, there are transient increases in myocardial wall thickness that presumably reflect myocardial edema and are correlated to the length of cold ischemic time, but not to the presence of rejection [51]. Previous work in pig models have shown significant myocardial edema resulting from the administration of cardio-protective solutions used during organ harvesting [52,53]. These findings suggest that T2 relaxation times are not able to discriminate rejecting and non-rejecting allografts in the peri-operative period due to normal inflammation and edema that occurs early after heart transplantation.

In a series of studies by Marie et al., it was found that T2 relaxation times were significantly higher in patients with International Society of Heart and Lung Transplant (ISHLT 1990) grade 2 or 3 rejection compared to those with less than grade 2 rejection (60 ms vs. 51 ms, p = 0.0001) [16,20]. The sensitivity in these studies was 89% and specificity was 75% and 91%, respectively [16,20]. Marie et al. also demonstrated that T2 relaxation times normalized following treatment of the rejection episode (60 ms vs. 49 ms) [16]. In a subgroup analysis[16], patients categorized as 'false positive' (i.e. elevated T2 relaxation time with no biopsy evidence of rejection) were significantly more likely to develop rejection in the subsequent three months than patients with both normal CMR and normal biopsy results (Sensitivity 63%, Specificity 78%).

Any assessment of diagnostic accuracy is predicated on the accuracy of the 'Gold Standard'. The utility of endomyocardial biopsy is clear, however, its diagnostic accuracy is not well characterized and is essentially impossible to study in a human population. The well recognized, and not uncommon, entity of biopsy negative rejection suggests that the sensitivity of endomyocardial biopsy is less than 100%. Therefore disagreements between CMR and biopsy may be a deficiency in CMR, endomyocardial biopsy or both. The subgroup analysis by Marie et al. that suggests that CMR might predict rejection in patients who are CMR 'positive' and biopsy negative raises the exciting possibility that CMR may be more sensitive than biopsy at detecting rejection.

##### Limitations to T2 imaging

Signal dropout and motion artefacts are well recognized problems affecting image quality in T2 weighted spin-echo techniques. These pulse sequence deficiencies are accentuated by low magnetic field strength, arrhythmia, and long scan times. Most of the papers in the field of T2 imaging and transplant rejection predate the newer and shorter Turbo Spin Echo (TSE) sequences and relied on the much slower spin echo techniques with long scan times. In addition many studies presented in this review obtained their images using magnetic field strength well below the current standard of 1.5 T, which yields lower signal to noise ratios. Long scan times and low field strength could both have had significant negative impact on image quality. TSE sequences reduce scan times, but T2 quantification can be adversely affected by stimulated echoes. TSE sequences are also sensitive to RF and static field inhomogeneities [54]. T2 weighted images are prone to signal gradation related to the proximity of the sampled area to the acquisition coil. This has been largely corrected with newer signal intensity correction algorithms, however some of the studies presented in this review used T2 weighted images that were likely subject to this potential error. Biological factors may also affect T2 quantification as many tissues types including the heart have been shown to have multi-exponential T2 behaviour [55]. Under sampling the T2 decay curve can lead to a two fold error in T2 estimation when multiexponential T2 behaviour is present[54].

He et al. have recently developed a T2 quantification sequence for the purpose of measuring myocardial iron in patients with thalassemia [56]. This novel spin-echo technique has since been installed and validated at four other international centres [57]. The coefficient of variation for local inter-study and inter-site variability was reported as 4.4% and 5.2%, respectively.

Despite the limitations of T2 weighted imaging mentioned above, previous work in transplant rejection studies has shown a consistent linear association between T2 relaxation times and the degree of acute transplant rejection. Furthermore, the relatively mild degree of rejection detectable with this technique (i.e. grade 2 ISHLT 1990) would not even require therapy in many contemporary transplant settings. This suggests that the relationship between T2 relaxation and rejection is highly sensitive and very unlikely to miss any cases of advanced rejection. Improvements in T2 imaging in the current era such as higher field strengths, fast TSE sequences, and improved blood and fat suppression techniques will likely strengthen the association between T2 relaxation times and transplant rejection[49].

### T1 weighted CMR

#### Myocardial T1 signal intensity

T1 weighted cardiovascular MR images are influenced by myocardial water content, although to a lesser extent than T2 weighted images. Non-contrast enhanced T1 signal intensity has shown an inconsistent correlation with rejection in animal models of heart transplantation [42,45]. Nishimura et al. used eight heterotopically transplanted dogs to show that T1 signal intensity was increased with rejection in a graded fashion that correlated with the degree of rejection [45]. Similar trials did not confirm these results [29,42]. Revel et al. assessed the utility of T1 signal intensity to diagnose rejection in 29 human heart transplant recipients and found no significant correlation with rejection as diagnosed by endomyocardial biopsy [38].

#### Myocardial T1 quantification

The T1 relaxation time can be calculated from a series of images acquired with an increasing delay following an inversion or saturation of the magnetization. A longer time of recovery is represented by larger T1 values, and like T2 values, this typically reflects an environment of fewer restrictions to water motion, such as edema. Aherne et al. and Nishimura et al. used a dog model to show that T1 relaxation times are prolonged in rejecting hearts compared to non-rejecting hearts [29,45]. Animal spectroscopy studies have also shown a significant prolongation of T1 relaxation times with rejection [30,40].

Wisenberg et al. investigated 25 human heart transplant recipients and found that mean T1 relaxation times were significantly prolonged for those with rejection compared to non-rejectors (497 ± 30 ms vs. 360 ± 21 ms, p < 0.05) [33]. In this study, both T1 and T2 relaxation times were only correlated with biopsy proven rejection after 24 days post transplantation. As with T2 relaxation time, T1 relaxation time within the first 24 days post transplantation are presumably influenced by perioperative factors unrelated to rejection.

The relationship between T1 relaxation and transplant rejection has been less well studied than that of T2 relaxation. The superior sensitivity to water content of T2 weighted imaging makes it a better choice for imaging myocardial inflammation, and likely accounts for the paucity of trials investigating T1 relaxation and rejection.

### T1 Contrast agents

#### Gadolinium

##### Early Enhancement

Gadolinium based contrast agents are by far the most common contrast agents used in clinical CMR imaging. Intravenous gadolinium increases signal intensity on T1 weighted images acquired early after contrast administration, in proportion to the degree of tissue perfusion and is thought to reflect the hyperemia seen in inflamed tissue. Increase in signal intensity early after contrast injection (early enhancement) has shown utility in the diagnosis of other disorders of myocardial inflammation such as myocarditis [26]. Abdel-Aty et al. found that early enhancement alone was too insensitive for diagnosing myocarditis, but was useful when used in combination with T2 values and late gadolinium enhancement in a scoring system for the diagnosis of myocarditis [26]. Yoshida et al. used a non-working allograft heart transplant model in dogs to demonstrate a 25–42% increase in signal intensity post gadolinium in rejecting allografts compared to native hearts [58]. Konstam et al. showed that T1 weighted maximal myocardial signal intensity post gadolinium infusion could identify three distinct grades of rejection in a rat model [59].

In two human trials of transplant rejection, post contrast signal intensity tended to increase with degree of rejection although it could not consistently identify the full spectrum of abnormal endomyocardial biopsies diagnostic of rejection [35,36]. Alemnar et al. tested several variables of contrast enhanced myocardial signal intensity in 40 heart transplant patients and found no association with rejection[35]. Mousseaux et al. examined 39 heart transplant patients for an association between biopsy proven rejection and myocardial enhancement (post contrast myocardial SI – pre contrast myocardial SI/pre contrast myocardial SI) within ten minutes post gadolinium injection [36]. They found an increase in myocardial enhancement in rejectors compared with non-rejectors (mean enhancement: grade 1 rejection = 70 +/- 14%, grade 2 or 3 rejection = 81 +/- 27%, non-rejectors 53% +/- 24, p < 0.05). However, myocardial enhancement was not able to discriminate rejection severity [36].

##### Ventricular Wall Thickness and Systolic function

The correlation between biopsy proven rejection and echocardiographically determined ventricular morphology is specific in severe cases of acute cellular rejection but is too insensitive to be used as a screening tool [60]. It has been postulated that the superior spatial resolution of CMR may lead to improved sensitivity in diagnosing rejection on the basis of changes to ventricular morphology [23].

Myocardial wall thickness has been shown to increase in both animal [41,61] and human [33,38] CMR trials of transplant rejection. Several animal studies showed that increased wall thickness during acute rejection was correlated to ex-vivo total myocardial water content [29,30,40,62]. Wall thickness was not capable of accurately identifying the severity of a rejection episode.

Revel et al. studied 29 heart transplant patients using CMR and found that wall thickness increased during acute rejection and decreased as the rejection episode resolved [38]. Wisenberg also showed that left ventricular wall thickness was increased in patients with rejection (ISHLT ≥ grade 2) compared to those without rejection (21 mm vs. 13 mm) [33]. Alemnar et al. performed CMR on 40 transplant patients receiving contemporary anti-rejection therapies and found no significant differences in ventricular volume, wall thickness, and ejection fraction between those with and without histologic evidence of rejection [35]. In the late 1990s, animal trials by Yoshida and Walpoth found that hearts undergoing rejection had reduced ejection fraction and stroke volume, although these changes were only significant when rejection was moderate or severe [61,62].

Changes in ventricular morphology and systolic function as measured by CMR are associated with rejection [60,63,64]. Despite the excellent spatial resolution of CMR, these variables are probably of insufficient sensitivity to detect the early and milder forms of rejection that are of clinical interest.

### Potential CMR Correlates of Heart Transplant Rejection

#### Diastolic Dysfunction

Diastolic dysfunction is one of the earliest measurable features of heart transplant rejection [65]. Yoshida et al. used a working heart model of untreated, syngenic and allogenic heart transplants in rats to assess left ventricular end diastolic pressure volume relationship (LVEDPVR) [62]. Invasive catheterization was used to modulate cardiac preload and measure pressures. CMR was used to assess ventricular volumes. These data were then compiled into LVEDPVR curves for various time points during rejection. At four days post transplant, the untreated allograft group showed a significant reduction in compliance compared to the isograft group. The reduction in compliance preceded any evidence of systolic dysfunction. Despite these provocative results, there have been no human studies assessing CMR measures of diastology in Transplant rejection. Measuring diastolic function with CMR may improve sensitivity in diagnosing rejection, however work in this area would need to differentiate changes in diastolic properties due to rejection and those due to the fibrotic and hypertrophic remodeling that accompanies heart transplantation even in the absence of rejection[66].

#### Twisting Mechanics

Left ventricular twisting mechanics have also been studied in normal and transplanted hearts. Using magnetic resonance tagging, Donofrio et al. found that non-rejecting pediatric transplanted hearts had normal strain measurements, but abnormal torsion patterns compared to normal hearts[67]. There were no episodes of rejection in this study, thus differences between rejectors and non-rejectors could not be assessed.

Hansen et al. used implanted radio-opaque intramyocardial markers and biplane fluoroscopy to serially study twist and untwist in 12 heart transplant recipients [68]. They found a 25% decrease in torsional deformation amplitude and peak systolic torsion during periods of rejection compared to pre-rejection values. Despite validated techniques for CMR to quantify myocardial strain and torsion, there have been no trials correlating CMR measures of twisting mechanics and transplant rejection.

#### Late Gadolinium Enhancement

Gadolinium can also be used in CMR to detect areas of myocardial scar or myocardial fibrosis. The rate at which gadolinium is cleared from the myocardium is slower in areas with fibrosis compared to healthy myocardium. T1 weighted images taken several minutes ("late") after contrast injection will show higher concentrations of gadolinium in areas of myocardial fibrosis making these areas appear bright. Late gadolinium enhancement (LGE) has correlated well to pathologic assessment of myocardial fibrosis in ischemic [69,70] and non-ischemic [26,71,72] myocardial injury. A recent study of LGE patterns in heart transplant patients found that 50% of patients had a non-ischemic LGE pattern similar to that seen in diseases of myocardial inflammation such as myocarditis[73]. No study to date has looked at presence, degree, or location of LGE patterns in acute human heart transplant rejection.

### T1 and T2 Contrast Agent

#### Iron oxide particles

Iron oxide contrast agents have been used in clinical and experimental MR since the 1980s predominantly in the field oncology [74-76]. More recently, these agents have been shown to be safe [77] and useful for contrast MR angiography[78,79]. Iron oxide contrast agents contain superparamagnetic particles with an iron oxide crystal core wrapped in an outer coating (i.e. dextran) which shorten both T1 and T2/T2* relaxation [80]. Over time, iron oxide particles are taken up by macrophages which shortens their T2/T2* properties. Thus, accumulation of macrophages, which contain iron oxide, in inflamed tissue can be visualized as a signal loss on T2 weighted images.

Kanno et al. showed that T2 signal intensity decreased 24 hours after iron oxide particle injection in untreated rat allografts compared to isografts at day seven (SI: 95% vs. 70%) [81]. Signal intensity in rejecting allografts returned to baseline after treatment with cyclosporine for seven days. Immunohistochemistry confirmed accumulation of iron oxide containing macrophages in areas of rejection [81].

Iron oxide contrast agents have also been used in a rat model of cardiac transplant rejection to study hyperemia. Immediately after injection, iron oxide particles remain intravascular unless there are alterations in local vascular permeability as seen in inflamed tissue. Extravasation of iron oxide particles leads to an increase in signal intensity in these regions on T1 weighted images. Johansson et al. showed that six days post transplant, T1 signal intensity was increased in untreated rat allografts compared to isografts within 5 minutes of iron oxide injection (mean difference 25%)[82]. Penno et al. used a T1 weighted 3-D spoiled gradient echo sequence to show that myocardial signal intensity in rejecting rat allografts was significantly elevated compared to immunosuppressed allografts within four minutes post contrast injection[83]. Treatment of the rejection episode reversed the increase in signal intensity. The rapidity of the change in signal intensity suggests altered vascular permeability is responsible for the increase in signal.

CMR with iron oxide particles is a novel and potentially powerful method to evaluate inflammation in the heart. T1 Imaging early post iron oxide contrast injection can identify increased vascular permeability, while delayed T2 imaging gives information into in-vivo macrophage accumulation. Human trials of transplant rejection and iron oxide contrast agents are needed.

## Conclusion

Several CMR variables have shown good correlation to biopsy proven heart transplant rejection, the strongest of which is quantitative T2 assessment. Criticism regarding the reproducibility of T2 measures[84] as well as limited access to CMR have likely hampered the adoption of CMR into routine post transplant clinical care. Improvements in CMR hardware combined with appropriate pulse sequences for T2 quantification makes routine ascertainment of T2 relaxation more feasible [56,57] and improves inter-center reproducibility over traditional T2 results based on signal intensity. Early enhancement may also prove useful in diagnosing transplant rejection just as it has in the diagnosis of myocarditis. Studies are needed to evaluate promising CMR correlates of rejection such as diastolic function, ventricular twist, late gadolinium enhancement, and paramagnetic iron oxide contrast agents. Future studies should focus on combining multiple CMR measures into a transplant rejection scoring system to improve the sensitivity in detecting heart transplant rejection and possibly reduce, if not eliminate, the need for endomyocardial biopsy.

## Competing interests

The authors declare that they have no competing interests.

## Authors' contributions

CB contributed to design of the review and manuscript preparation. RT contributed to design of the review and manuscript preparation. MH contributed to design of the review and manuscript preparation. MT contributed to manuscript preparation. IP contributed to design of the review and manuscript preparation.
